# Reducing Dental Sealant Disparities in School-aged Children Through Better Targeting of Informational Campaigns and Public Provision of Sealants [Response to Letter]

**Published:** 2005-09-15

**Authors:** Kari Jones

**Affiliations:** National Center for Health Marketing, Division of Public–Private Partnerships, Centers for Disease Control and Prevention, Atlanta, Ga

## In Reply:

On behalf of my coauthors and myself, I would like to thank Dr Bolin for initiating a discussion of dental sealants (1) and encouraging us to expand our comments beyond those conveyed in our abstract (2). The Centers for Disease Control and Prevention promotes school-based and other publicly provided sealant campaigns, because it is recognized that children from lower-income families often lack access to, and means to pay for, dental care. These programs generally target low-income children; most of these school-based programs have minimum thresholds for percentage of the school's students on free and reduced-cost lunch. Where they exist, these programs have been successful at sealing teeth among low-income children and reducing disparities; we found no significant difference in the nationwide prevalence of sealants among low-income white, black, and Hispanic children. However, many of these programs' administrators have reported both having difficulties gaining access to schools and missing opportunities to seal the teeth of a significant number of children because of unreturned permission slips. Thus, we suggest that educating school administrators and parents about the benefits of dental sealants will increase participation in public programs, thereby decreasing dental health disparities in children. We agree with Dr Bolin's point and hope to see more public support for school sealant programs targeting low-income children.
